# AI-clinician collaboration via disagreement prediction: A decision pipeline and retrospective analysis of real-world radiologist-AI interactions

**DOI:** 10.1016/j.xcrm.2023.101207

**Published:** 2023-09-27

**Authors:** Morgan Sanchez, Kyle Alford, Viswesh Krishna, Thanh M. Huynh, Chanh D.T. Nguyen, Matthew P. Lungren, Steven Q.H. Truong, Pranav Rajpurkar

**Affiliations:** 1 Department of Biomedical Informatics, Harvard Medical School, Boston, MA 02115, USA; 2 Department of Computer Science, Columbia University, New York, NY 10027, USA; 3 Department of Computer Science, Stanford University, Stanford, CA 94301, USA; 4 VinBrain JSC, Hanoi 11622, Vietnam; 5 VinUniversity, Hanoi 12450, Vietnam; 6 Microsoft Corporation, Redmond, WA 98052, USA; 7 University of California San Francisco, San Francisco, CA 94143, USA; 8 Stanford University, Stanford, CA 94301, USA

**Keywords:** human-AI collaboration, computer-aided diagnosis, clinical decision support, machine learning, artificial intelligence, radiology, clinician workload estimation, disagreement prediction, AI safety

## Abstract

Clinical decision support tools can improve diagnostic performance or reduce variability, but they are also subject to post-deployment underperformance. Although using AI in an assistive setting offsets many concerns with autonomous AI in medicine, systems that present all predictions equivalently fail to protect against key AI safety concerns. We design a decision pipeline that supports the diagnostic model with an ecosystem of models, integrating disagreement prediction, clinical significance categorization, and prediction quality modeling to guide prediction presentation. We characterize disagreement using data from a deployed chest X-ray interpretation aid and compare clinician burden in this proposed pipeline to the diagnostic model in isolation. The average disagreement rate is 6.5%, and the expected burden reduction is 4.8%, even if 5% of disagreements on urgent findings receive a second read. We conclude that, in our production setting, we can adequately balance risk mitigation with clinician burden if disagreement false positives are reduced.

## Highlights

• A diagnostic model alone may not suffice for effective AI-clinician collaboration
• Post-deployment data enable improvement upon existing pipelines
• Modeling diagnoses, disagreement, significance, and confidence may reduce burden

## Graphical abstract

**Figure:**
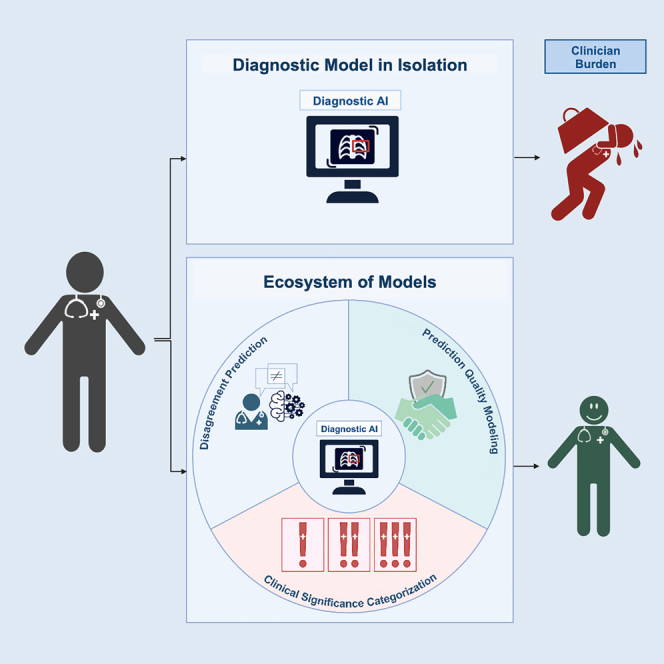

## Teaser

A diagnostic model alone may not suffice for effective AI-clinician collaboration, but post-deployment data enable improvement upon such pipelines through simulation. Sanchez et al. make use of post-deployment data and propose a pipeline that integrates diagnosis, disagreement, significance, and confidence modeling to promote safer AI-clinician collaboration and reduce clinician burden.

## Introduction

In recent years, significant strides in artificial intelligence (AI) for medicine have led to the integration of AI models into clinical workflows in a number of areas. From screening tools for diabetic retinopathy^1^^,^^2^ and colon cancer^3^^,^^4^ to diagnostic aids for pulmonary nodules^5^ and tuberculosis,^6^ AI for clinical applications is becoming increasingly popular.^7^

AI-based decision support tools typically aim to accomplish two major goals: improve patient care and reduce provider cost and burden. Encouragingly, numerous studies present evidence that AI support can increase diagnostic performance or reduce diagnostic variability over clinicians working without AI support.^4^^,^^5^^,^^8^ Previous work has also demonstrated instances of reduced clinician workload in AI-assisted workflows.^9^ However, these potential benefits do not come without risks.^10^ We know, for example, that AI can fail in any deployment setting for a number of reasons, including distribution shift between training and deployment and underperformance in particular subpopulations,^11^^,^^12^ and it is especially important to mitigate these issues in clinical settings, where the stakes are high and poor predictions can cause harm to patients. Alarmingly, low-quality predictions can actually degrade performance, misleading even the most experienced clinicians.^13^ Therefore, to mitigate the consequences of poor AI performance, these models are often used in an assistive setting, where a final decision is still made by a clinician. This allows for clinicians to correct mistakes made by the AI, but it still puts a heavy burden on the user, even in straightforward cases where the AI and the clinician are likely to agree. In addition, simply providing the clinician with all predicted diagnoses equivalently, regardless of accuracy or urgency, may limit the effectiveness of the tool and introduce dangerous biases. For instance, over time, clinicians may begin to rely too heavily on the tool and default decisions to the model, even in circumstances where there may otherwise be doubt as to the accuracy of the result. This is known as automation bias. On the other hand, clinicians may begin to ignore recommendations from the tool if they find themselves sorting through excessive false positives. Otherwise, they may miss important diagnoses, distracted by overwhelming numbers of predictions to assess. This phenomenon is similar to what is known as alert fatigue and is another example of how AI assistance integration can fail even if the model itself is performing relatively well.

To better understand where AI-clinician collaboration could improve and determine how to best protect against these risks, we analyze real-world production data from a chest X-ray interpretation aid deployed in Vietnam hospitals and study cases of AI-clinician disagreement. Inspired by these real-world data, we hypothesize that we can incorporate protections against common AI safety issues while simultaneously reducing or maintaining clinician burden in this production setting. In particular, we can use production data to predict disagreement, and motivate decisions as to when and how to present model output using characteristics such as the expected trustworthiness of a given prediction and whether the clinician is expected to agree with it along with the clinical significance of each pathology. What follows is a decision pipeline for AI-supported diagnosis that is expected to mitigate risks of AI-assistance while maintaining or reducing clinician burden.

## Results

### Disagreement in real-world production data

The data used in this work were collected in a post-deployment setting of DrAid, an AI-supported chest X-ray interpretation tool. DrAid employs an AI model that takes in a chest X-ray image and outputs the presence or absence of 21 pathologies and findings. In the interface, the radiologist is presented with (1) the patient’s medical and Rx history, (2) a chest X-ray in DICOM format, (3) relevant demographics such as age and gender, (4) predicted pathologies and other findings, and (5) regions of interest (ROIs) associated with each AI-predicted finding. The user can view ROIs, add AI findings to a report, request a second opinion, search for additional pathologies to add, and generate both internal and patient-accessible reports.

Data used for disagreement analysis and decision pipeline ideation and simulations consisted of predicted pathologies, final radiologist report inclusions, and demographics for 10,569 patients. Patients were randomly sampled from those whose X-rays were interpreted using DrAid at Nam Dinh Hospital in Vietnam between October of 2019 and February of 2022. Furthermore, to ensure high-quality samples, only images interpreted by a radiologist whose familiarity with the DrAid tool was in the top 10, as measured by number of images interpreted using the tool, were included in the study. Pathologies and findings detected include cardiomegaly, fracture, lung lesion, pleural effusion, pneumothorax, atelectasis, consolidation, pneumonia, edema, cavitation, fibrosis, enlarged cardiomediastinum, widened mediastinum, pleural other, medical device, COVID-19, mass, nodule, mass or nodule (unknown), lung opacity, and tuberculosis (see Table 1 for definitions). COVID-19, however, was excluded from the analysis due to lack of usage in the DrAid workflow, and enlarged cardiomediastinum was excluded due to overwhelming redundancy with cardiomegaly and widened mediastinum. One additional category, “other findings,” was also excluded from analysis due to lack of specificity.

**Table 1:** Descriptive statistics for DrAid pathologies

Finding	Prevalence (%)	Disagreement rate (%)	Diagnostic TPR (%)	Diagnostic FPR (%)
Atelectasis	5.99	7.12	83.11	6.50
Cardiomegaly	2.37	1.86	97.84	1.85
Cavitation	1.23	6.14	72.50	5.87
Consolidation	1.73	2.18	97.63	2.18
Edema	0.08	0.08	62.50	0.05
Fracture	2.38	1.68	91.42	1.51
Lung lesion	0.76	0.55	85.14	0.44
Lung opacity	0.00	0.00	0.00	0.00
Mass	0.78	1.36	89.47	1.29
Mass or nodule	4.88	9.16	64.36	7.80
Medical device	0.79	1.12	80.52	0.97
No finding	42.08	26.60	55.00	13.23
Nodule	10.75	2.64	95.24	2.38
Pleural effusion	5.73	2.63	83.21	1.77
Pleural other	17.65	11.04	82.61	9.68
Pneumonia	0.10	0.10	0.00	0.00
Pneumothorax	1.38	1.20	93.33	1.12
Pulmonary scar	18.07	15.35	98.02	18.29
Tuberculosis	11.81	4.16	76.69	1.60
Widening mediastinum	2.23	11.84	83.49	11.73
Average	6.54	5.34	74.60	4.41
Prevalence-weighted average	–	–	76.36	4.06
Median (IQR)	2.30 (0.79–7.59)	2.41 (1.18–6.90)	83.16 (70.47–91.90)	1.81 (1.08–6.35)

In addition to removing several findings from the study, 794 samples were removed due to lack of radiologist labels, leaving 9,775 patients in the final study population. Following the aforementioned preprocessing steps, the vast majority of samples (6,730) contained only a single finding (including those in the “no finding” category), 151 samples had no label, and 1,111 samples had 2 labels. Approximately 1,500 samples total had between 3 and 5 labels, but under 150 total samples had more than 6. In addition, one radiologist annotated the vast majority (>99%) of the samples.

The average and median rates of disagreement between the final radiologist label and the diagnostic model label for the Nam Dinh Hospital sample (independent of the predicted ROIs) were 6.5% and 2.6%, respectively (IQR, 1.3–9.6). The prevalence-weighted average true positive rate (TPR) and false positive rate (FPR) of DrAid over all pathologies using the final report as ground truth were 76.4% and 4.1%, respectively. Medians were 83.2% (IQR, 70.5–91.9) and 1.8% (IQR, 1.1–6.4). Further descriptive statistics for each finding are included in Table 1. These estimates, especially the low disagreement rate, inspired a pipeline that uses predicted agreement to organize predictions and protect against biases introduced by using an AI-based interpretation aid.

### Incorporating clinical significance, disagreement modeling, and AI quality modeling into presentation of predictions

In the following section, we walk the reader through an anecdotal example illustrating how clinicians might interface with the proposed pipeline, first describing an example interaction, and then going on to describe all components of the pipeline and how they interact in detail. For illustrative purposes, imagine that a patient enters the emergency department following an automobile accident. The patient is experiencing sharp chest pain and shortness of breath, and the heart rate is elevated. Following the initial assessment, a chest X-ray is ordered. The radiology department at this institution employs an AI-supported chest X-ray interpretation interface, which allows radiologists to process and finalize reports efficiently and safely by steering attention toward the most pressing pathologies.

**Figure 1:**
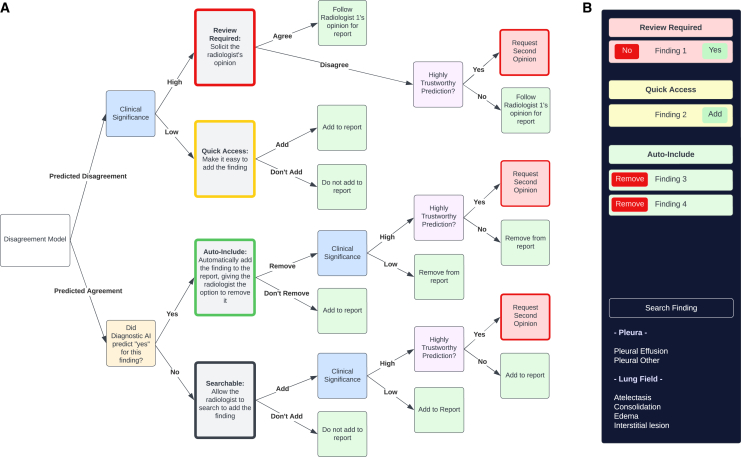
Proposed decision pipeline and sample interface (A) Proposed decision pipeline. The initial four categories are represented in order of required attention, where findings are sorted based on the Disagreement Model, Diagnostic Model, and Clinical Significance Classification output. Later in the pipeline, clinician action as well as prediction quality and clinical significance drive requests for a second opinion. (B) Sample interface for the proposed pipeline. For demonstration purposes, all of the top 3 categories are shown in the sample interface, but it is unlikely that all have findings in them.

The interpretation tool interface appears as follows: the X-ray occupies the majority of the screen, and a small bar appears on the right-hand side, which enables the radiologist to add and remove findings from the report (see Figure 1). For the present patient, the first section in this bar contains one finding, pneumothorax, along with associated options, “Yes” and “No.” Behind the scenes, the diagnostic AI model did not find pneumothorax, but the system predicted disagreement, causing this finding to be listed at the top of the screen for easy access and to intelligently protect against automation bias. It is important to note, however, that although one finding is listed in this section for this sample interaction, in practice the first section is rarely populated, since disagreement is so uncommon in our dataset. Observing pneumothorax, the radiologist adds it to the report and moves on to the following section.

The second section also contains only one finding with expected disagreement: medical device. Findings in this section, however, are expected to require less immediate intervention, so the radiologist must only interact with the interface if the decision is made to add it to the report. The radiologist explores the AI-predicted ROI, observes a small artifact that may have been mistaken for a pacemaker, but does not add this finding to the report. The final section is the “Auto-Include” section. Here, “Fracture” is listed with an accompanying option to remove it. Observing a small rib fracture, the clinician takes one last pass through the X-ray and submits the report, marking the end of an approximately 30–60 s process that results in a highly structured, queryable report. The system instills additional assurance by automatically requesting a second opinion in rare instances of disagreement on severe findings where the system is highly confident in the AI model’s prediction. The final report is sent back to the emergency department, where the patient is treated for pneumothorax and provided analgesics for a rib fracture.

The proposed decision pipeline attempts to simultaneously accomplish three overarching goals. First, it assigns priority to findings based on expected disagreement and clinical significance and communicates that priority to the radiologist. Second, it attempts to automate inclusion or exclusion of findings from the report while minimizing risk of adverse effects and, finally, it attempts to improve report accuracy by automatically requesting a second opinion in rare cases of predicted uncertainty.

Concretely, to make use of disagreement, clinical significance, and AI quality modeling to inform when and how to present AI findings, the pipeline requires four primary components.

1. Diagnostic model: a model that predicts pathologies (e.g., based on a patient’s chest X-ray).
2. Disagreement model: a model that predicts whether the radiologist will agree or disagree with the AI prediction for a particular pathology.
3. Clinical significance categorization: a categorization that reflects clinical significance for each potential pathology. It is used to assign elevated priority to more urgent indications.
4. Prediction quality model: for a particular diagnostic model prediction, a model that assigns a class regarding the trustworthiness of that prediction (e.g., either high or low).

Pathologies (i.e., findings) are assigned to one of four categories: Review Required, Quick Access, Automatic Inclusion, and Searchable. These categories are presented to a radiologist in order, as they are organized by decreasing demand for radiologist attention. Note, however, that findings in the Searchable category are not explicitly listed for review.

The “Review Required” category is for *high clinical significance* findings with *predicted disagreement*. Before submitting a report, the user is required to select “yes” or “no” for each of these findings. The “Quick Access” category, on the other hand, is for *low clinical significance* findings also with *predicted disagreement*. The user is expected to, at least, view these findings and add any at their discretion, but interaction is not necessary for report submission. For the “Automatic Inclusion” category, the description is quite self-explanatory. These are findings predicted by the Diagnostic Model to be *present in the image* that have *predicted agreement*, and the user is only presented with the option to “remove” each finding. Finally, the “Searchable” category includes findings that the Diagnostic Model predicts are *not present in the image* for which there is also *expected agreement*. The user may search for the finding to add it to the report.

For all categories, if the user ultimately disagrees with an AI prediction classified as trustworthy or “high quality” by the Prediction Quality Model and “highly significant” as per the clinical significance classification, the finding is automatically sent for a second opinion. Although not the focus of this study, we anticipate that second readings would be best implemented such that readers are not biased by AI or human input. Therefore, we envision the question to the second reader as a simple “Does this patient have x pathology?” to avoid biasing the reader toward AI or human output. This also protects against alert fatigue since the reader is provided with both positive and negative examples. A visual representation of this pipeline and an example interface is presented in Figure 1.

### Decision pipeline structure design process

It is important to note that the proposed decision pipeline structure is not the only reasonable way to organize the four models to create a decision tree. The pipeline design process involved extensive discussions about key design considerations such as safety, clinician burden, and clinician attention. For each of the 16 combinations of characteristics (since each of the 4 models outputs 2 possible values), we determined what we would expect from a safe and cost-efficient pipeline in terms of whether and how to present model output. The decision tree was created by combining similar examples and determining a clear visual representation. For example, through discussions, it was determined that, in cases of disagreement, to take advantage of clinical domain expertise and keep the approach clinician centered, clinical significance of the pathology, as opposed to estimated prediction quality, should be the primary determining factor for how much attention a clinician gives to that pathology. To guide clinician attention, we employ two techniques: placing pathologies in a natural order of priority from top to bottom and disallowing report submission for “Review Required” pathologies. Although it would have made sense to incorporate diagnostic model output in conjunction with prediction quality into this priority classification, we opted for a simpler approach and one that is less likely to bias the user by displaying AI output in cases of expected disagreement.

### Expectations and considerations for the proposed pipeline

The effect on clinician workload and report accuracy of adding Disagreement, Clinical Significance, and Prediction Quality models to a diagnostic aid workflow as described above is dependent on a number of factors. For instance, auto-including findings may reduce clinician burden, but auto-requesting a second opinion, although potentially useful for augmenting report accuracy, may increase that burden. An important question to ask is: What is the overall effect on clinician burden, and what might contribute to that value? In the following analysis, we simulate burden for a variety of disagreement model performance levels and prediction quality model characteristics, but there are likely other factors to consider as well.

### Burden comparison

**Figure 2:**
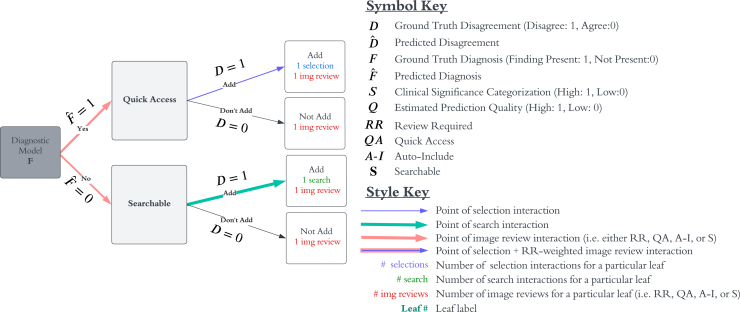
Baseline pipeline interactions and key Pathologies are sorted into “Quick Access” and “Searchable” categories based on Diagnostic Model output (i.e., $\hat{F}$) in the baseline pipeline. Colored arrows highlight sources of interaction for a particular edge with higher weights indicating larger cost associated with the interaction. The baseline workflow has two types of image review interactions (Quick Access and Searchable) as well as interactions associated with selecting “add” next to a finding (i.e., a selection interaction) or searching for a finding by typing it into a search box (i.e., a search interaction).

**Figure 3:**
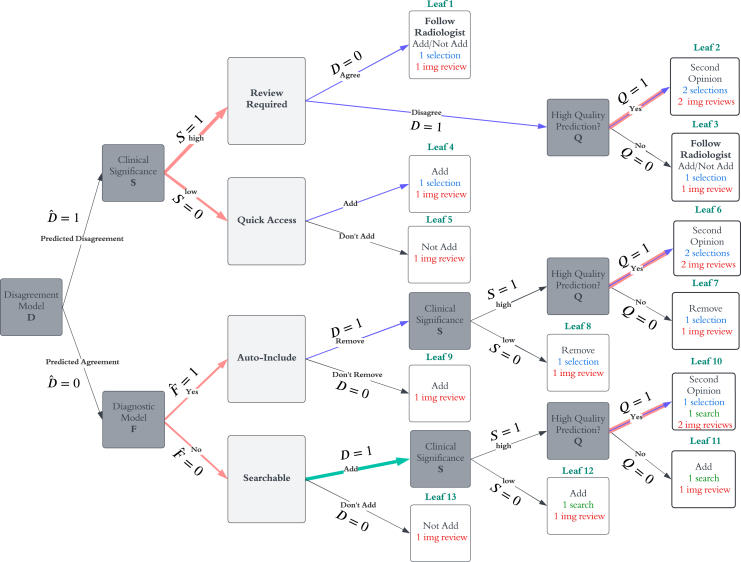
Proposed decision pipeline interactions Like the baseline pipeline diagram, colored arrows highlight sources of interaction for a particular edge with higher weights indicating larger cost associated with the interaction. The proposed pipeline has four types of image review interactions as well as interactions associated with selecting “add,” “remove,” “yes,” or “no” (i.e., a selection interaction) or searching for a finding by typing it into a search box (i.e., a search interaction). Please refer to the key in Figure 2 to interpret colors and symbols and refer to Table 2 for weights associated with each type of interaction.

To estimate the theoretical overall effect on clinician workload, we compare the proposed pipeline to a baseline workflow, where a Diagnostic Model similar to the DrAid tool sorts findings into the “Quick Access” and “Searchable” categories, displaying all findings that the AI predicts are present to the user. A diagram of such a baseline pipeline is shown in Figure 2. In particular, to compare clinician burden, we assign a cost to each potential user interaction in each of the pipelines and sort each finding from each patient in the Nam Dinh data into the appropriate leaves in both pipelines. Next, we determine the proportions of the data in each leaf and weight each leaf by total expected interactions. Finally, we sum these weighted proportions to obtain overall interaction burden for each pipeline and calculate the ratio of proposed pipeline burden to baseline burden. In assigning interaction cost, we assign selection weight to selection interactions such as “yes,” “no,” “add,” or “remove,” search weight to findings added from the Searchable section, and image review weight to account for viewing the X-ray to consider that particular finding. For simplicity, we assign a weight of 1 to selection interactions and a weight of 4 to search interactions. Image review weight is assigned such that higher weight is applied to higher-priority findings (i.e., Review Required > Quick Access > Auto-Include > Searchable), and such that the average expected weight in the baseline pipeline is about 30, which we expect to equate to approximately 30 s, but further data collection is required to obtain the most accurate weights. Weights used in this analysis (Table 2) are merely reasonable estimates and are not data driven. They are currently modeled as suggestions to the user (e.g., we would like the user to spend 4 times as much time on each review required finding compared with each searchable one) and may not reflect actual time spent on each interaction. See Figures 2 and 3 for interaction cost details in both baseline and proposed pipelines.

**Table 2:** Definitions and default values used for burden simulations

	Selection weight	1
	search weight	4
W_S_	searchable image review weight	1
W_A-I_	auto-include image review weight	1.5
W_QA_	quick access image review weight	3.5
W_RR_	review required image review weight	4
TPR_*dis*_	default disagreement model TPR	0.8
FPR_*dis*_	default disagreement model FPR	0.03
P*sec_read*	default probability that a second opinion is requested for a clinically significant finding with AI-clinician disagreement	0.05

For the proposed pipeline, however, we are still missing three important pieces of information in the Nam Dinh data: clinical significance labels, prediction quality labels, and disagreement model predictions. For clinical significance, we assign a single label to each pathology represented in the Nam Dinh data with the help of experts (see Table S1), but we recognize that clinical significance is likely context dependent. For example, if an X-ray is requested to confirm placement of a support device, the “Medical Device” should not be placed in the “low clinical significance” category. Although we use one static categorization for our analysis, in a deployment setting, categorizations could be fluid, with the clinical indication informing finding placement.

In addition, since we do not have ground truth prediction quality labels and because the accuracy of such labels will not affect burden in our analysis (only prevalence of positive predictions), we simulate second opinion requests at a variety of probabilities by drawing from Bernoulli distributions. Finally, because we aim to study the effect of different disagreement models on burden, we simulate disagreement model predictions for a variety of TPRs and FPRs. For samples with and without AI-clinician disagreement, we again draw from Bernoulli distributions with probabilities of predicting disagreement equal to the TPRs and FPRs, respectively.

### How do prediction quality and disagreement model performance affect burden?

**Figure 4:**
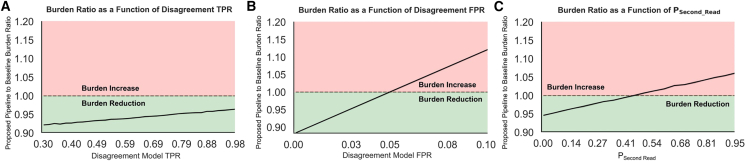
Clinician burden simulations (A) The effect of varying TPR for the disagreement model on theoretical clinician burden, fixing the other two parameters using the default values in Table 2. (B) The effect of varying FPR for the disagreement model. (C) The effect of varying the probability that a clinically significant finding with AI-clinician disagreement receives a second opinion. For all plots, the green-shaded portion demonstrates characteristics that reduce clinician burden over baseline, whereas red-shaded areas represent increased burden. All lines are plotted with 95% confidence intervals, although uncertainty is very low, so they are not obviously visible.

To better understand how clinician burden is affected by characteristics of the prediction quality and disagreement models, we estimate the ratio of expected proposed pipeline burden to baseline burden for a range of second reading probabilities as well as disagreement model TPRs and FPRs, varying one quantity at a time and otherwise using the default values listed in Table 2. To account for stochasticity in the Bernoulli trials and patient populations, we repeat each experiment 10 times, each time sampling 9,775 patients with replacement. Visualizations for each of the simulations are shown in Figure 4.

We find that, as the TPR increases from 0.3 to 0.95, the ratio stays relatively constant increasing only from 92.218% ± 0.001% to 96.464% ± 0.001%. This constitutes a slightly reduced burden for the baseline and proposed pipelines with a larger reduction for low TPRs. Burden ratio is much more sensitive, however, to FPR. Although an FPR of 0.01 can result in an expected 9.55% ± 0.06% reduction in burden, an FPR of 0.10 actually results in a 12.04% ± 0.09% increase. Perhaps most interesting, however, is the effect of adjusting the probability that a second opinion is requested for cases of disagreement on clinically significant findings. Even if all cases of disagreement on clinically significant findings receive a second read, burden is only increased by 6.67% ± 0.07%. If, however, second reads are restricted to less than 5% (e.g., only the most trustworthy AI predictions), burden is reduced by 4.8% ± 0.1%. Combining all of these, if the disagreement model TPRs and FPRs are 0.8 and 0.03, respectively, and the conditional probability of a second read is 0.05, burden is reduced by 4.8% ± 0.2%.

## Discussion

### Why? Understanding trends in burden comparison

Following simulation experiments, we decided to dig into exactly why burden is changed, why it is so sensitive to the disagreement model’s FPR, and why it is less sensitive to the disagreement model’s TPR and the conditional probability of a second reading. The primary components of the proposed pipeline that differentiate it from baseline are the addition of the “Review Required” and “Auto-Include” sections as well as the ability to automatically and intelligently request second opinions, each of which have differing effects on clinician burden and some of which even provide additional safeguards for report accuracy.

The “Review Required” section includes two types of findings: (1) clinically significant findings that the diagnostic model predicts are present, but that we expect the radiologist to disagree with, and (2) clinically significant findings that the diagnostic model did not find, but that the radiologist should still consider due to expected disagreement. One might expect that forcing radiologists to interact with all of these findings might increase clinician burden, and, for some models and deployment settings, this may be true. This is actually the source of the large slope in Figure 4B. As the FPR increases, data points move primarily from leaf 13 to leaf 1, resulting in one additional selection interaction per finding as well as a higher image weight. Because of the low prevalence of each pathology in our dataset, if the disagreement model FPR is too high, the second category will include a large number of irrelevant findings. Disagreement, however, in our dataset is also very uncommon, so if we can predict it with a low FPR, very few findings will actually fall into these categories. Furthermore, the first category of findings is already included in the list of AI findings presented to the radiologist in the baseline pipeline, so this is not a source of additional burden. We justify adding the second category, however, for ease of access and as a safeguard to automation bias. In the baseline pipeline, the radiologist would need to identify this finding without AI assistance and then search for it, but in the proposed pipeline it is included in the list of findings to consider, hopefully reducing bias toward pathologies the diagnostic model is best at finding. Ultimately, the risk associated with a high FPR is very important to consider when choosing a disagreement model and pipeline, but it comes with the added benefit of protection from automation bias and reduction in findings that need to be manually searched for and added to the report when used appropriately.

The second large difference between the pipelines is the Auto-Include category. Again, although it is not unreasonable to hypothesize that auto-including findings could contribute to increased burden as a result of radiologists spending excessive amounts of time removing extraneous findings from the report, we do not find this to be the case in our simulations. We instead find that, for the DrAid model, each pathology is so uncommon and disagreement is so rare that, especially if you can predict disagreement with a high TPR, cases of disagreement with an auto-included finding are very rare. Auto-including findings, however, without accounting for cases of expected disagreement or using diagnostic models with high FPRs may result in increased burden, so, again, it is important to consider this when choosing models.

Finally, perhaps the most potentially burdensome addition to the pipeline is auto-requesting a second opinion. However, the proposed pipeline restricts this action to clinically significant findings with AI-clinician disagreement. Therefore, because disagreement is so rare in our dataset, even though many of the findings are marked as significant, very few second opinions are requested, even when we set P_sec_read_ as high as 1.

### How characteristics of the dataset may affect results

**Figure 5:**
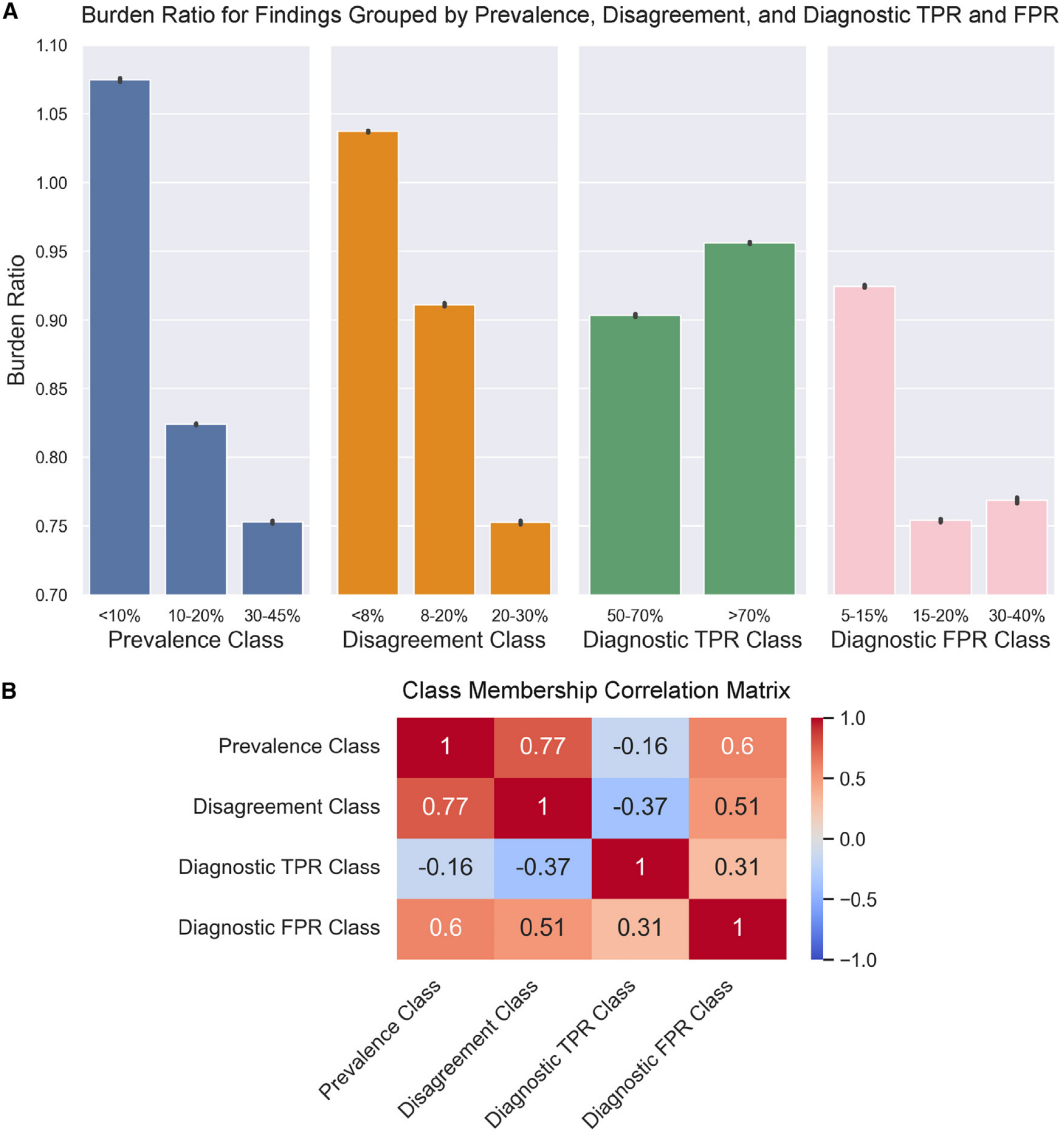
Burden ratio and finding characteristics (A) Burden ratio for findings stratified on prevalence, disagreement rate, diagnostic model TPR, and diagnostic model FPR; 95% confidence intervals are shown in black. (B) Pearson correlation coefficients for prevalence, disagreement rate, diagnostic TPR, and diagnostic FPR classes.

To further examine generalizability of results and study how different characteristics of findings in the dataset impact burden, we pursue a secondary analysis on the effects of prevalence, disagreement rate, diagnostic model TPR, and diagnostic model FPR on burden. To accomplish this, we create two to three classes for each characteristic that reflect natural breaks in the distributions and compute burden ratios for just the findings in that class when disagreement model TPRs and FPRs are 0.8 and 0.03, respectively, and the conditional probability of a second read is 0.05. We chose to remove findings with 0 FPR or 0 TPR for their respective experiments since those are likely due to extremely low prevalence. Results from this analysis are included in Figure 5A, and we have also included a correlation matrix for these classes in Figure 5B. Prevalence, disagreement rate, and diagnostic FPR are all positively correlated with each other, with prevalence and disagreement being the most highly correlated. Diagnostic TPR is relatively uncorrelated with the other characteristics with slight correlations in both positive and negative directions.

We observe similar largely downward trends as prevalence, disagreement rate, and diagnostic FPR increase, but this is understandable due to the high correlation between these characteristics. Therefore, unless these correlations hold in the new setting, it is difficult to use this analysis to predict relative burden ratio in new settings using our production data alone. We, therefore, do not suggest implementing the proposed pipeline in a new setting without repeating burden analysis on production data from the setting of interest. However, for similar settings, we might expect that higher average prevalence, disagreement rate, and diagnostic FPR might result in reduced burden.

### Limitations of the study

Here, we show that we can use disagreement and clinical significance models in conjunction with a diagnostic model to guide clinician interaction with AI predictions in our production setting. This alternative to using the diagnostic model alone can result in minimal changes or even slight reductions in clinician burden, while providing additional AI safety measures.

This study, however, has limitations. For example, this analysis was performed using one dataset from one institution, and we were not able to study the separate effects of important quantities such as prevalence of disagreement and pathologies as well as performance of the diagnostic model as correlations between these characteristics were very high. We also did not account for clinician experience level when making decisions about when and how to present model output and in analyzing results, although previous work has shown that the experience level of a clinician or user can impact the effectiveness and risks of the AI assistance.^13^ Furthermore, although we explored expected effects on burden, we hypothesize that this proposed pipeline may also positively affect report accuracy and clinician experience over traditional AI assistance workflows. In future work, we hope to obtain ground truth labels for a subset of this dataset as well as data from other institutions and models to simulate the effects of these important quantities and characteristics on both burden and report accuracy.

In addition, to have control over TPR and FPR separately, we simulate predicted disagreement as a weighted coin flip. However, with this method, we do not capture important biases that we know AI models are often susceptible to, and instead the TPR and FPR are constant for all findings and samples. To remedy this, we could train a disagreement model and adjust prediction thresholds to obtain predictions for a variety of possible TPR and FPR pairs. The same could be done for the diagnostic and prediction quality models. This method, however, would reduce flexibility to study TPRs and FPRs outside of our tested values, but it may produce more accurate burden estimates.

For this analysis, the Prediction Quality Model was also simulated using a weighted coin flip because we did not have access to ground truth Diagnostic Model labels. This, of course, is not an intelligent model, and second reviews would be almost completely random if not for the clinical significance categorization. Ideally, we would use a model that tells us how much we should trust a particular prediction. One simple way to do this for a well-calibrated model is using AI confidence. That is, if we know the model outputs probabilities close to 1 for findings that are most likely present in the image and close to 0 for findings likely not present in the image, we might be able to use distance from 0.5 as a proxy for prediction quality, or “trustworthiness” of a particular prediction. Although calibration is model agnostic, it can be more difficult to calibrate complex models. Thus, other measures of confidence may be necessary depending on the difficulty of achieving a well-calibrated model. In addition, because we did not obtain multiple reads on the same image to determine when an AI-clinician disagreement was the result of a faulty prediction or a controversial diagnosis, we did not build a Prediction Quality model, but this is a potential direction for future work.

Finally, another major limitation of our burden estimation analysis lies in the determination of interaction costs. Although we made use of reasonable estimates for selection and search weights, and informed image review weights with the overall goal of achieving a reasonable baseline burden in seconds, ratios of image review costs between the four categories (Review Required, Quick Access, Auto-Include, and Searchable) merely reflect suggested weights of each of these categories. Even if we provide suggestions to users, however, actual review time may be different. Future work should involve human-computer interaction experiments which estimate actual interaction cost of each path in the pipelines.

### Conclusion

In this work, we discuss a number of limitations and considerations for deploying AI-assisted diagnostic aids. We study disagreement in real-world production data from a chest X-ray interpretation tool and use that data to motivate the ideation of an AI assistance pipeline. We detail this pipeline, which makes use of machine learning to intelligently decide when and how to present model output in a clinically conscious manner, and again use the production data to simulate its effect on clinician burden depending on the characteristics of two models in the pipeline.

We found that clinician burden can be maintained, or even reduced, despite introducing additional safety measures by thinking beyond the traditional AI-assisted diagnostic workflow, using models of disagreement, clinical significance, and prediction quality to guide presentation of diagnostic model predictions to the clinician. We note, however, that burden is not the only outcome that requires rigorous validation, and we intend to perform a separate human-computer interaction study that directly validates burden estimates and quantifies the effects of the intended AI safety protections. We find, in simulation, using our real-world data, that burden is dependent on a number of characteristics such as disagreement model TPR and the fraction of findings sent out for a second opinion, but it is most sensitive to FPR of the disagreement model. For burden to be decreased, however, the disagreement model must have a fairly small FPR, and this is important to consider when choosing an operating point and AI-assistance pipeline.

Although results of this work are not intended to directly generalize to new settings and tools, and the scope of particular burden reduction values are specific to our dataset, through this work we hope to inspire researchers to consider alternatives to boosting underlying model performance as they look to enhance AI-supported diagnostic workflows. We also hope to encourage future work that uses production data to estimate burden and other relevant measures of a tool’s success. This could lead to choices of models with optimal characteristics informed by relevant outcome estimates as opposed to typical machine learning evaluation metrics.

## STAR★Methods

### Key resources table

**Table:** 

REAGENT or RESOURCE	SOURCE	IDENTIFIER
**Deposited data**		

Raw Data	this paper	Mendeley Data: https://doi.org/10.17632/ck27s2h85n.2
Source Data	this paper	https://github.com/morgsmss7/RadAI_Usage

**Software and algorithms**		

Python 3.7.3	Python	RRID:SCR_008394
Seaborn 0.11.2	Seaborn	RRID:SCR_018132
NumPy 1.21.6	NumPy	RRID:SCR_008633

### Resource availability

#### Lead contact

Further information and requests for resources should be directed to and will be fulfilled by the lead contact, Morgan Sanchez (MorganSanchez@g.harvard.edu).

#### Materials availability

This work did not produce new unique reagents.

### Experimental model and subject details

This study did not involve human subjects. All analyses were retrospective analyses of previously de-identified data, and it was obtained by the authors without patient intervention. The authors had no interactions with the persons represented in the data nor with any identifiable private information.

### Method details

#### DrAid data exclusions

Pathologies and findings detected by the diagnostic model include cardiomegaly, fracture, lung lesion, pleural effusion, pneumothorax, atelectasis, consolidation, pneumonia, edema, cavitation, fibrosis, enlarged cardiomediastinum, widened mediastinum, pleural other, medical device, COVID-19, mass, nodule, mass or nodule (unknown), lung opacity, and tuberculosis (see Table S1 for definitions). COVID-19, however, was excluded from the analysis due to lack of usage in the DrAid workflow, and enlarged cardiomediastinum was excluded due to overwhelming redundancy with cardiomegaly and widened mediastinum. The “other findings” label was also excluded from analysis due to lack of specificity. Further, 794 samples were removed due to lack of radiologist labels.

### Quantification and statistical analysis

Python (v3.7.3) was used to analyze the data and produce visualizations, plots include 95% confidence intervals via Seaborn’s built in error bar functionality for barplots and lineplots, and correlation coefficients are computed using the Pearson method.

For pipeline simulations, outputs from the Disagreement Model and the Prediction Quality Model are sampled from Bernoulli distributions via NumPy. Probabilities of success are TPR_d_ or FPR_d_ for the Disagreement model, depending on whether the sample is marked as positive or negative for each finding, and P_sec_read_ for the Prediction Quality Model. To account for this stochasticity, burden ratios are computed 10 times by sampling the study population with replacement.

## Data Availability

• Tabular data have been deposited at Mendeley Data and are publicly available as of the date of publication. Accession numbers are listed in the key resources table. • All original code has been deposited at GitHub and is publicly available as of the date of publication. DOIs are listed in the key resources table. • Any additional information required to reanalyze the data reported in this paper is available from the lead contact upon request.

## Supplemental Information

Document S1. Table S1

Document S2. Article plus supplemental information
