# Supplementary material for: AI-clinician collaboration via disagreement prediction: A decision pipeline and retrospective analysis of real-world radiologist-AI interactions
Source: Cell Rep Med. 2023 Sep 27;4(10):101207. doi: 10.1016/j.xcrm.2023.101207 (PMC10591030; doi:10.1016/j.xcrm.2023.101207)
Supplement: Document S1. Table S1 [file mmc1.pdf]

Cell Reports Medicine, Volume *4*

Supplemental information

AI-clinician collaboration via disagreement prediction: A decision pipeline and retrospective analysis of real-world radiologist-AI interactions

Morgan Sanchez, Kyle Alford, Viswesh Krishna, Thanh M. Huynh, Chanh D.T. Nguyen, Matthew P. Lungren, Steven Q.H. Truong, and Pranav Rajpurkar

Supplemental Items

DrAid™ Finding	Definition	Clinically Significant?
Atelectasis	Decrease in the volume of the lung or part of the lung.<br>Atelectasis consists of several types:<br>· Obstructive pulmonary atelectasis or absorption atelectasis due to obstruction in the lumen of bronchi<br>· Cicatrisation atelectasis due to parenchymal fibrosis<br>· Relaxation atelectasis or passive atelectasis caused by extrinsic compressions such as pleural effusion or pneumothorax<br>· Adhesive atelectasis due to diffuse surfactant deficiency	No
Cardiomegaly	There is no single definition for cadiomegaly, and the size of cardiac silhouette is relative to body weight, height, surface area, age, and sex. There are many studies on the correlation between the size of cardiac silhouette and cardiothoracic ratio measurements. DrAid™ detects an enlarged cardiothoracic ratio (i.e., greater than about 50%), calculated by dividing the sum of maximum distances from the centerline to two sides of the heart contour by the largest inside diameter (excluding ribs) of the chest.	Yes
Cavitation	A cavity is detected. In DrAid™, a cavity is defined as any radiographic opacity with an internal area of lucency, regardless of wall thickness.	Yes
Consolidation	Alveoli are filled with fluid, blood, cells, tissue, or other substances.	Yes
Edema	Drainage of fluid from the blood vessels into the interstitial tissue and alveoli of the lung.<br>There are two types, namely edema due to cardiovascular causes and non-cardiovascular causes. Edema may progress over many stages, or there may only be accumulated fluid in the interstitial tissue in early stages without diffuse alveolar damage, so edema is not a subset of air space opacification	Yes
Fracture	A partial or complete discontinuity in a bone is seen on the chest X-ray	Yes
Lung Lesion	This encompasses all images labeled as containing a mass, nodule, or cavitation.	Yes
Lung opacity	Conditions detected in which the alveoli, airways, and interstitial tissue are replaced by substances such as fluid, blood, cells and bacteria, leading to a decrease in the ratio of gas to soft tissue. This definition covers a wide variety of lesions which can be divided into the following types: air space opacification, linear opacification, and nodular opacification	Yes
Mass	A pulmonary opacity lesion of over 30 mm in diameter	Yes
Mass or Nodule	A pulmonary opacity lesion of unspecified diameter	Yes
Medical Device	A medical instrument or device used in patient support or treatment is detected. EKG and oxygen wires are not labeled.	No

No finding	No abnormalities are detected in the chest x-ray	No
Nodule	An opacity lesion, usually round, either clearly restricted or not, ≤ 3 cm in diameter	Yes
Other Findings	A lesion is detected which does not match other definitions	Yes
Pleural effusion	The presence of "fluid" in the pleural space is estimated to be above the normal limit of 15 ml.	Yes
Pleural other	Refers to findings such as pleural thickening, pleural calcification, and pleural tumors	No
Pneumonia	An inflammatory disease of lung "parenchyma" characterized by alveoli and/or interstitial tissue lesions. In DrAid™ context, pneumonia includes:<br>· Lobar pneumonia<br>· Bronchopneumonia (must incorporate information of age if the patient is a child)<br>· Interstitial lung disease	Yes
Pneumothorax	An abnormal presence of gas in the pleural space.	Yes
Pulmonary Scar	Overgrowth, hardening, and/or scarring of various tissues can be attributed to excess deposition of extracellular matrix components including collagen. It is the end result of chronic inflammatory reactions induced by a variety of stimuli including persistent infections, autoimmune reactions, allergic responses, chemical insults, radiation, and tissue injury.	No
Tuberculosis (TB)	Chest x-ray warrants reasonable suspicion of an infectious disease caused by Mycobacterium tuberculosis. Tuberculosis can be found in all parts of the body, but pulmonary tuberculosis, the type that DrAid™ detects, is the most common form. It should be noted that the specificity of TB diagnosis via x-rays is not high, so diagnosis of pulmonary tuberculosis should not be made based on only one chest x-ray film	Yes
Widened Mediastinum	No single precise quantitative measurement was used for this finding because it is highly dependent on techniques, ages, and clinical pictures. Doctor experience greatly informs sensitivity, so labels in this data reflect this variability.<br>Common clinical pictures:<br>· Abnormal widening of blood vessels originating from the heart: aortic aneurysm, aortic enlargement, enlarged pulmonary artery.<br>· Suspected mediastinal tumors, mediastinal lymph nodes.<br>· Diaphragmatic hernia, etc.	Yes

Table S1. Definitions and Clinical Significance Categorizations for DrAid™ Pathologies, Related to STAR Methods.
